# Comparative transcriptome profiling of susceptible and tolerant citrus species at early and late stage of infection by “*Candidatus* Liberibacter asiaticus”

**DOI:** 10.3389/fpls.2023.1191029

**Published:** 2023-06-14

**Authors:** Chenying Gao, Cuixiao Li, Ziyi Li, Yaoxin Liu, Jiaming Li, Jun Guo, Jiana Mao, Fang Fang, Cheng Wang, Xiaoling Deng, Zheng Zheng

**Affiliations:** ^1^ National Key Laboratory of Green Pesticide, South China Agricultural University, Guangzhou, Guangdong, China; ^2^ Guangdong Province Key Laboratory of Microbial Signals and Disease Control, South China Agricultural University, Guangzhou, China; ^3^ Horticulture Research Institute, Guangxi Academy of Agricultural Sciences, Nanning, Guangxi, China; ^4^ Institute of Tropical and Subtropical Cash Crops, Yunnan Academy of Agricultural Sciences, Baoshan, Yunnan, China

**Keywords:** Huanglongbing, *Candidatus Liberibacter asiaticus*, tolerance, transcriptomics, host response

## Abstract

Citrus Huanglongbing (HLB), caused by “*Candidatus* Liberibacter asiaticus” (CLas), is the most destructive disease threatening global citrus industry. Most commercial cultivars were susceptible to HLB, although some showed tolerant to HLB phenotypically. Identifying tolerant citrus genotypes and understanding the mechanism correlated with tolerance to HLB is essential for breeding citrus variety tolerance/resistance to HLB. In this study, the graft assay with CLas-infected bud were performed in four citrus genotypes, including *Citrus reticulata* Blanco, *C. sinensis*, *C. limon*, and *C. maxima*. HLB tolerance was observed in *C. limon* and *C. maxima*, while *C. Blanco* and *C. sinensis* were susceptible to HLB. The time-course transcriptomic analysis revealed a significant variation in differentially expressed genes (DEGs) related to HLB between susceptible and tolerant cultivar group at early and late infection stage. Functional analysis of DEGs indicated that the activation of genes involved in SA-mediated defense response, PTI, cell wall associated immunity, endochitinase, phenylpropanoid and alpha-linolenic/linoleic lipid metabolism played an important in the tolerance of *C. limon* and *C. maxima* to HLB at early infection stage. In addition, the overactive plant defense combined with the stronger antibacterial activity (antibacterial secondary and lipid metabolism) and the suppression of pectinesterase were contributed to the long-term tolerance to HLB in *C. limon* and *C. maxima* at late infection stage. Particularly, the activation of ROS scavenging genes (catalases and ascorbate peroxidases) could help to reduce HLB symptoms in tolerant cultivars. In contrast, the overexpression of genes involved in oxidative burst and ethylene metabolism, as well as the late inducing of defense related genes could lead to the early HLB symptom development in susceptible cultivars at early infection stage. The weak defense response and antibacterial secondary metabolism, and the induce of pectinesterase were responsible for sensitivity to HLB in *C. reticulata* Blanco and *C. sinensis* at late infection stage. This study provided new insights into the tolerance/sensitivity mechanism against HLB and valuable guidance for breeding of HLB-tolerant/resistant cultivars.

## Introduction

Citrus Huanglongbing (HLB, also called citrus greening) is the most devastating disease for global citrus industry (Bové, 2006; Zheng et al., 2018). In China, HLB is caused by a phloem-limited unculturable Gram-negative alpha-proteobacteria, “*Candidatus* Liberibacter asiaticus” (CLas) (Bové, 2006). Citrus trees affected by HLB usually exhibited yellow shoots, mottling/yellowing of leaves, deformed fruit with color inversion, premature fruit abscission, aborted seeds, and ultimately the death of trees (Bové, 2006; Zheng et al., 2018). Management of HLB is difficult and expensive, particularly no cure is currently available for HLB. The recommended management practices for HLB in disease endemic regions in China mainly included the use of CLas-free nursery stocks, control of insect vectors and removal of infected trees (Zheng et al., 2018). Due to the lack of effective treatment for HLB-affected trees, the use of HLB resistant/tolerant varieties become one of the most effective approaches for the ultimate long-term HLB solution. Although most commercial citrus cultivars were susceptible to HLB (Bové, 2006), some were found to have potential tolerance to HLB (Folimonova et al., 2009). However, for breeding HLB-tolerant citrus varieties, it is essential to understand the differences in response of tolerant and susceptible citrus varieties to HLB.

To better understand the HLB tolerance mechanism in citrus, comparative transcriptomic profiling of susceptible and tolerant citrus varieties in response to CLas infection have been performed through microarray and high-through sequencing technologies (Albrecht and Bowman, 2008; Fan et al., 2012; Hu et al., 2017; Yu et al., 2017; Zou et al., 2019; Arce-Leal et al., 2020; Curtolo et al., 2020; Wei et al., 2021). Transcriptomic analysis revealed a stronger immune response in tolerant rough lemon (*Citrus jambhiri*) than in susceptible sweet orange (*Citrus sinensis*) during CLas infection (Yu et al., 2017). Genes involved in cell wall metabolism, secondary metabolism, signaling, transcription factors and redox reactions were contributed to the tolerance of Kaffir lime (*Citrus hystrix*) and Mexican lime (*Citrus aurantifolia*) to HLB (Hu et al., 2017; Arce-Leal et al., 2020). In addition, the Methyl salicylate signaling and its mediated systemic acquired resistance (SAR) response were also correlated with tolerance to HLB in sour pomelo (*Citrus grandis* Osbeck) (Zou et al., 2019). A wide-ranging transcriptomic analysis of *Poncirus trifoliata*, *Citrus sunki*, *Citrus sinensis* and three contrasting hybrids identified a specific genetic mechanism of HLB tolerance, including the downregulation of gibberellin (GA) synthesis and the induction of cell wall strengthening (Curtolo et al., 2020). A recent transcriptomic analysis showed that the HLB-tolerant Australian finger lime (*Citrus australasica*) had evolved specific redox control systems to mitigate the reactive oxygen species and modulate the plant defense response, and activated genes responsible for the production of Cys-rich secretory proteins and Pathogenesis-related 1 (PR1-like) proteins against CLas infection (Weber et al., 2022). Most recently, the anatomical study and the seasonal transcriptome profiling analysis of HLB-tolerant ‘LB8-9’ Sugar Belle^®^ mandarin (“Clementine” mandarin × “Minneola” tangelo) showed that the phloem regeneration contributed to the tolerance to HLB (Deng et al., 2019; Ribeiro et al., 2023). The substantial transcriptomic studies in response to CLas infection in tolerant citrus varieties suggested that despite some cellular and defense response being common among tolerant cultivars against CLas, others responses were cultivars/genotypes-specific.

With no effective cure currently available for HLB-affected citrus trees, breeding of HLB-tolerant citrus varieties become one of promising strategies to control HLB in an effective long-term solution. Therefore, studies are still necessary to better understand the differences of host response between susceptible and tolerant citrus cultivars, which can provide useful guidance for developing HLB-resistant/-tolerant varieties in the future. In this study, we performed a greenhouse rigorous assay in four different citrus genotypes (*Citrus reticulata* Blanco, *C. Sinensis*, *C. limon*, *C. maxima*) by grafting with CLas-infected buds and comparing the disease development in four cultivars, aiming to evaluate their tolerance to HLB among four cultivars. HLB tolerance was observed in *C. limon*, and *C. maxima*, while *C. reticulata* Blanco and *C. sinensis* showed sensitive to HLB. A time-course comparative transcriptomic study was further performed in four cultivars at early and late CLas infection stage to provide a comprehensive overview of host response against CLas infection and to reveal the molecular mechanisms of tolerance/susceptibility of different citrus genotypes in response to CLas/HLB.

## Materials and methods

### Plant material and experimental design

Two-year-old CLas-free seedlings of four citrus cultivars, including ‘Shatangju’ mandarin (*Citrus reticulata* Blanco cv. Shatangju, grafted on *Poncirus trifoliata*), ‘Hongjiang’ orange (*C. sinensis* cv. Hongjiang Cheng, grafted on *C. reticulata* Blanco cv. Hongju), ‘Eureka’ lemon (*C. limon* cv. Eureka, grafted on *Citrus jambhiri*), and ‘Shatian’ pomelo (*C. maxima* cv. Shatian Yu, grafted on *C. grandis*), were used as host sources for grafting with CLas-infected budwoods. All CLas-infected budwoods were collected from HLB-affected lemon trees (*Citrus limon*) located in Ruili city of Yunnan province and confirmed by CLas-specific Real-time PCR with primer set CLas4G/HLBr before using for grafting (Bao et al., 2020). For each cultivar, a total of 15 CLas-free two-year-old seedlings were grafted with CLas-infected budwoods and five others seedlings were grafted with budwood from healthy lemon plants as control (mock-grafted). After grafting, all plants were maintained in insect-proof greenhouse and fertilized as needed. The HLB symptom was recorded every four wpg. For each grafted plant, leaf samples (closed to grafting budwoods and from the new flush) were collected from each plant every 4 wpg. DNA was extracted from the leaf midribs and used for CLas quantification. For RNA-Seq analyses, six complete leaves, including three closed to grafting budwoods and three from new flush, were collected at 12 wpg and 48 wpg, respectively. Leave samples used for RNA-Seq analysis were immediately frozen with liquid nitrogen when sampling. Three biological replicates of CLas-infected citrus plants and two biological replicates of mock-grafted citrus plants were collected for RNA-Seq.

### DNA and RNA extraction

For DNA extraction, 100 mg of fresh leaf midrib tissue was cut into small section and grinded with MP FastPrep^®^-24 Grinder (MP Biomedicals LLC, Santa Ana, CA, U.S.A.) using speed of 4 M/S for 1 min. Total DNA was extracted by using the E. Z. N. A. HP Plant DNA Kit (OMEGA Bio-Tek Co., Guangdong, China) according to the manufacturer’s manual. For RNA extraction, the midribs (~250 mg) of six leaves from individual plant were dissected and mixed as one sample. Total RNA extraction was performed using E. Z. N. A. Total RNA Kit I (OMEGA Bio-Tek Co., Guangdong, China) following the manufacturer’s manual. The concentration of all extracted DNA and RNA samples were tested by Qubit 2.0 (Thermo Fisher Scientific Inc., Waltham, MA, U.S.A.). The quality of RNA samples was examined by Agilent 2100 (Agilent Technologies Inc., Santa Clara, CA, U.S.A.) before sequencing.

### Quantification of CLas

CLas quantification was performed by Real-time PCR with CLas-specific primer set (CLas4G: AGTCGAGCGCGTATGCGAAT/HLBr: GCGTTATCCCGTAGAAAAAGGTAG) and probe (HLBp: FAM-AGACGGGTGAGTAACGCG-BHQ) according to our previous study (Bao et al., 2020). TaqMan^®^ quantitative Real-time PCR were performed in CFX Connect Real-Time System (Bio-Rad, Hercules, CA). PCR mixture (20 μL) contained 10 μL of PerfectStart^®^ II Probe qPCR SuperMix (TransGen Biotech, Beijing), 1 μL of DNA template (~25 ng), 0.4 μL of each forward and reverse primer (10 μM), 0.2 μL of PCR Probe (10 μM) and 8 μL of ddH2O. The procedure of PCR included incubation at 95°C for 2 min followed by 40 cycles of amplification (95°C for 10 s and 58°C for 30 s, with fluorescence signal capture at the end of each 58°C step). PCR result (Ct value) was obtained by using Bio-Rad CFX Manager 2.1 software with automated baseline settings and threshold. DNA Sample with Ct value less than 34 was considered as CLas positive.

### RNA sequencing and transcriptomic analysis

The library preparation for RNA-Seq was constructed with a NEBNext^®^ Ultra™ RNA Library Prep Kit for Illumina (New England Biolabs, Ipswich, MA, USA). The high-throughput sequencing was performed on an Illumina HiSeq 3000 system with output of 150-bp paired-end reads by the Novogene Company (Beijing, China). All clean HiSeq data from each sample were mapped to *Citrus sinensis* reference genome (GCA_022201045.1) (Wang et al., 2021) by using Tophat2 with the mismatch penalty of no more than two nucleotides (Kim et al., 2013). Reads number mapped to each gene was counted by using HTSeq v0.61 (Anders et al., 2015) and RPKM (Reads Per Kilobase of exon model per Million mapped reads) was calculated based on the length of the gene and reads count mapped to this gene. Differentially expressed genes (DEGs) between CLas-infected sample and mock-grafted control sample were identified by DEGseq (Wang et al., 2010) with the cut-off values setting as Log2 Fold change ≥│1│and q-value < 0.005. Gene Ontology (GO) enrichment analysis of all satisfied DEGs was implemented by the GOseq R package (Young et al., 2010). GO terms with corrected P-value less than 0.05 were considered significantly enriched by differential expressed genes. The statistical enrichment of differential expression genes in KEGG pathways was performed by using KOBAS 2.0 (Xie et al., 2011).

### Gene expression validation

To validate result of DEGs identified from RNA-Seq analyses, a total of 15 DEGs involved in cell wall metabolism, secondary metabolism, hormone-related pathways, lipid metabolism, plant-pathogen interaction and antioxidant activity were selected for reverse transcription (RT) qPCR analysis. All primer sets were listed in **Supplementary Table S12**. The same set of RNA samples used for RNA-Seq analyses were used for RT-qPCR. The first strand cDNA was synthesized using TransScript^®^ One-Step gDNA Removal and cDNA Synthesis SuperMix (TransGen Biotech, Beijing) according to the manufacture’s protocol. The RT-qPCR was performed in CFX Connect Real-Time System (Bio-Rad, Hercules, CA). PCR mixture (20 μL) contained 10 μL of TransStart^®^ Green qPCR SuperMix (TransGen Biotech, Beijing), 1 μL of cDNA template, 0.4 μL of each forward and reverse primer (10 μM) and 8 μL of ddH_2_O. The procedure of PCR included incubation at 95°C for 30 s followed by 40 cycles of amplification (95°C for 10 s and 60°C for 30 s, with fluorescence signal capture at the end of each 60°C step). The *Glyceraldehyde-3-phosphate dehydrogenase* (*GAPDH*) gene was selected as the internal reference control. The relative expression levels of selected genes were calculated by 2^-ΔΔCt^ method (Livak and Schmittgen, 2001). For each sample, the Log_2_ fold change was obtained from the ratio of the relative expression value of CLas-infected plant vs. mock-grafted control. For each gene, the Log_2_ fold change of RT-PCR was compared with the RNA-Seq analyses from the same sample (**Supplementary Figure S1**).

## Results

### Symptom development and bacterial quantification analysis of citrus plants after inoculation with CLas

**Figure 1:**
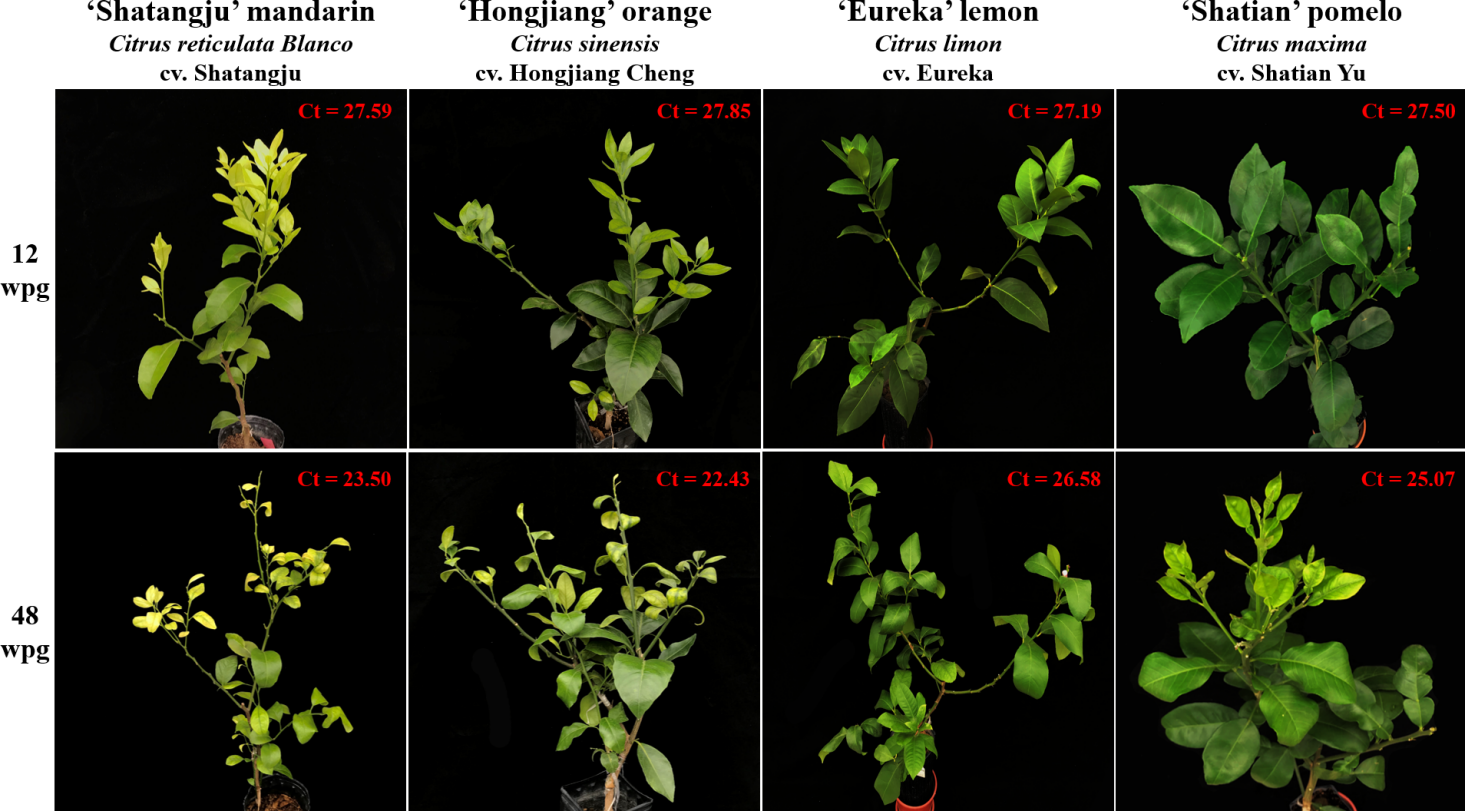
The representative infected plants of four citrus cultivars selected for RNA-Seq analysis after 12 and 48 weeks post-grafting (wpg). The Ct value was obtained with primer-probe set CLas4G/HLBp/HLBr.

qPCR result showed that CLas was first detected in four cultivars at 12 weeks post-grafting (wpg) (**Figure 1**), including six ‘Shatangju’ mandarin (40%), seven ‘Hongjiang’ orange (46.7%), four ‘Eureka’ lemon (26.7%) and eight ‘Shatian’ pomelo (53.3%). However, except one ‘Shatangju’ mandarin plant and one ‘Hongjiang’ orange plant showed early HLB symptoms (i.e. slight leaf yellowing) in new flush, others CLas-infected citrus plants were asymptomatic and phenotypically similar to the mock-grafted citrus plants at 12 wpg (**Figure 1**). All plants of four cultivars grafted with CLas-infected buds were confirmed to be CLas-positive at 20 wpg (**Table 1**). The typical HLB symptoms (i.e. blotchy mottling and yellowing of leaves) gradually exhibited in CLas-infected ‘Shatangju’ mandarin and ‘Hongjiang’ orange plants after 20 wpg. All infected ‘Shatangju’ mandarin plants and ‘Hongjiang’ orange plants showed HLB symptoms (yellowing/mottling leaf) at 32 and 36 wpg, respectively (**Table 1**). However, only four CLas-infected ‘Shatian’ pomelo plants and three ‘Eureka’ lemon plants showed leaf yellowing or zinc deficiency-like symptoms by the end of the study (48 wpg), although all ‘Eureka’ lemon and ‘Shatian’ pomelo plants had been identified as CLas-positive at 20 wpg (**Table 1**).

**Table 1:** “*Candidatus* Liberibacter asiaticus” detection result and Huanglongbing symptoms record of grafted citrus plants.

Cultivars	Detection result and symptoms record*	4 wpg	8 wpg	12 wpg	16 wpg	20 wpg	24 wpg	28 wpg	32 wpg	36 wpg	40 wpg	44 wpg	48 wpg
*Citrus reticulata* Blanco cv. Shatangju	Positive plants/Total plants	0/15	0/15	6/15	15/15	15/15	15/15	15/15	15/15	15/15	15/15	15/15	15/15
	Ct value	NA	NA	28.09	25.33	23.23	23.38	24.77	25.04	26.45	25.7	26.01	24.53
	Symptomic plants/Total plants	AS	AS	S (1/15)	S (3/15)	S (6/15)	S (9/15)	S (11/15)	S (14/15)	S (15/15)	S (15/15)	S (15/15)	S (15/15)
*Citrus sinensis* cv. Hongjiang Cheng	Positive plants/Total plants	0/15	0/15	7/15	14/15	15/15	15/15	15/15	15/15	15/15	15/15	15/15	15/15
	Ct value	NA	NA	27.53	25.35	21.37	20.56	24.2	25.71	26.31	25.92	24.55	24.76
	Symptomic plants/Total plants	AS	AS	S (1/15)	S (2/15)	S (5/15)	S (9/15)	S (12/15)	S (15/15)	S (15/15)	S (15/15)	S (15/15)	S (15/15)
*Citrus limon* cv. Eureka	Positive plants/Total plants	0/15	0/15	4/15	11/15	15/15	15/15	15/15	15/15	15/15	15/15	15/15	15/15
	Ct value	NA	NA	26.34	25.2	25.59	25.16	25.6	28.03	28.13	29.12	27.93	28.45
	Symptomic plants/Total plants	AS	AS	AS	AS	S (2/15)	S (2/15)	S (3/15)	S (3/15)	S (3/15)	S (3/15)	S (4/15)	S (4/15)
*Citrus maxima* cv. Shatian Yu	Positive plants/Total plants	0/15	0/15	8/15	10/15	15/15	15/15	15/15	15/15	15/15	15/15	15/15	15/15
	Ct value	NA	NA	28.12	28.07	25.22	28.79	26.64	27.37	27.4	26.47	27.27	26.43
	Symptomic plants/Total plants	AS	AS	AS	AS	AS	AS	AS	S (1/15)	S (1/15)	S (1/15)	S (2/15)	S (3/15)

*Ct values = the average Ct value of all CLas-positive plant. NA, No amplification; AS, Asymptomatic; S, Symptomatic. The number of HLB symptomatic plants are listed in the bracket. Leaf midribs sample collected at 12 wpg and 48 wpg were used for RNA-Seq.

Quantification result showed a similar proliferation pattern of CLas among four cultivars, i.e. the CLas concentration initially increased, then slightly decreased and maintained at a certain level in grafted citrus plants (**Table 1**). However, the concentration of CLas in infected ‘Shatangju’ mandarin and ‘Hongjiang’ orange was higher (with lower Ct value) than those observed in ‘Eureka’ lemon and ‘Shatian’ pomelo at same infection stage after 20 wpg until to 48 wpg (**Table 1**). Considering the symptom development and CLas concentration in four cultivars, the ‘Shatangju’ mandarin and ‘Hongjiang’ orange were relatively sensitive to HLB, while the ‘Eureka’ lemon and ‘Shatian’ pomelo were more tolerance to HLB. To further analyze the host response of CLas infection between sensitive and tolerant citrus cultivars at different disease development stage, the CLas-infected leaf samples of four cultivars were collected at 12 wpg (early infection stage, mostly asymptomatic) and 48 wpg (late infection stage, mostly symptomatic) and used for RNA-Seq analyses (**Figure 1**). As control, the leaf samples from mock-grafted citrus plants at 12 wpg and 48 wpg were also selected (**Supplementary Figure S2**).

### Identification of differentially expressed genes

**Figure 2:**
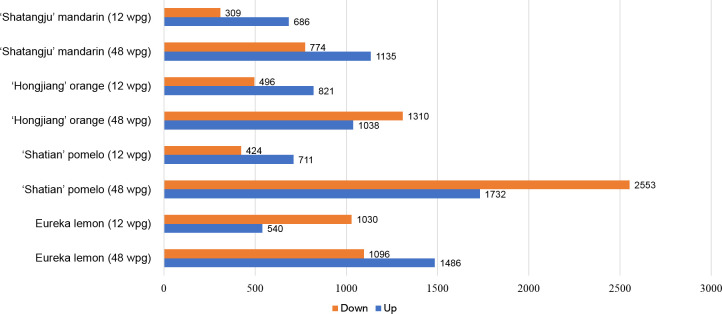
Statistic chart of differentially expressed genes (DEGs) of four citrus cultivars in response to “*Candidatus* Liberibacter asiaticus” infection at early (12 wpg) and late infection stage (48 wpg).

**Figure 3:**
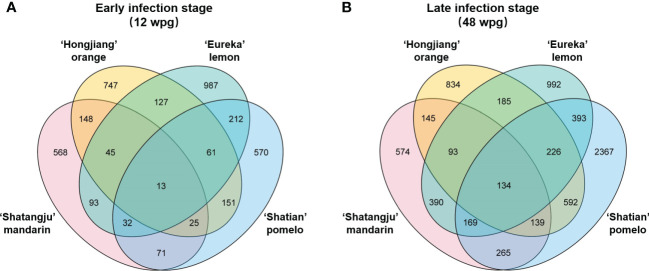
Venn diagram of differentially expressed genes (DEGs) among four cultivars in response to “*Candidatus* Liberibacter asiaticus” infection at early **(A)** and late infection stage **(B)**.

A total of 40 libraries were constructed and sequenced. The Illumina HiSeq platform generated a sequencing depth of 40~55 million 150 paired-end reads per library (Q30 > 93) (**Supplementary Table S1**). The biological replicates were highly correlated, with the Pearson’s correlation coefficient over 0.985 (**Supplementary Table S2**). Reference-based mapping to *Citrus sinensis* genome (GCA_022201045.1) showed that the ratio of mapped reads ranged from 88.17% to 93.47% for each HiSeq data (**Supplementary Table S2**). Overall, differential expression analysis identified a higher number of DEGs in late infection stage (48wpg) than those identified at early infection stage (12 wpg) in four cultivars when compared to the mock-grafted control (**Figure 2**). Notably, the DEGs number of ‘Shatian’ pomelo identified at 48 wpg was higher than those observed in other cultivars at 48 wpg (**Figure 2**). It was also found that two tolerant cultivars had more common DEGs than those identified in two susceptible cultivars at two infection stages (**Figure 3**). At 12 wpg, a total of 148 DEGs were commonly identified in susceptible ‘Shatangju’ mandarin and ‘Hongjiang’ orange and 212 DEGs were shared between tolerant ‘Eureka’ lemon and ‘Shatian’ pomelo (**Figure 3A**). A total of 145 common DEGs were identified between susceptible ‘Shatangju’ mandarin and ‘Hongjiang’ orange at late infection stage in compared to that of 393 common DEGs identified between tolerant ‘Eureka’ lemon and ‘Shatian’ pomelo (**Figure 3B**).

### Gene ontology and KEGG enrichment analysis of DEGs

GO assignments of DEGs identified a total of 19 and 29 significant enriched GO terms in susceptible ‘Shatangju’ mandarin and ‘Hongjiang’ orange at 12 wpg, respectively, while an increased number of significant enriched GO terms in tolerant ‘Eureka’ lemon and ‘Shatian’ pomelo was found to be 75 and 79 at 12 wpg, respectively (**Supplementary Table S3**). Thirty-eight significant enriched GO terms were commonly identified in ‘Eureka’ lemon and ‘Shatian’ pomelo at 12 wpg, mainly including hydrolase activity, response to stress, pyrophosphatase activity, nucleoside-triphosphatase activity, molecular function regulator, cell wall organization or biogenesis (**Supplementary Figure S3**, **Supplementary Table** **S4**). At 48 wpg, a total of 23, 64, 30, 47 significant enriched GO terms were identified in ‘Shatangju’ mandarin, ‘Hongjiang’ orange, ‘Eureka’ lemon and ‘Shatian’ pomelo, respectively (**Supplementary Table S3**). Five common significant enriched GO terms, including O-methyltransferase activity, cellular carbohydrate metabolic process, apoplast, extracellular region, and xyloglucan:xyloglucosyl transferase activity, were found in ‘Shatangju’ mandarin and ‘Hongjiang’ orange, while three significant enriched GO terms (transferring acyl groups, transferring acyl groups other than amino-acyl groups, terpene synthase activity) were commonly identified in ‘Eureka’ lemon and ‘Shatian’ pomelo at 48 wpg (**Supplementary Figure S3**, **Supplementary Table S3**).

KEGG pathways enrichment of DEGs showed that the MAPK signaling pathway, phenylpropanoid biosynthesis, plant hormone signal transduction, starch and sucrose metabolism, plant-pathogen interaction, amino sugar and nucleotide sugar metabolism, cysteine and methionine metabolism were mainly altered in four cultivars at two infection stages (**Supplementary Table S5**). More significant pathways were identified in four cultivars at 48 wpg than those observed at 12 wpg (**Supplementary Table S5**). The plant-pathogen interaction pathway and sesquiterpenoid and triterpenoid biosynthesis pathway were only significantly enriched in tolerant ‘Eureka’ lemon and ‘Shatian’ pomelo at 48 wpg (**Supplementary Table S5**). Especially, more DEGs were upregulated on the plant-pathogen interaction pathway in ‘Eureka’ lemon and ‘Shatian’ pomelo at 48 wpg (**Supplementary Table** **S5**). To further identify the DEGs that related to susceptibility and tolerance of citrus in response to HLB, the categories of DEGs related to disease response in susceptible cultivars group (‘Shatangju’ mandarin and ‘Hongjiang’ orange) and tolerant cultivars group (‘Eureka’ lemon and ‘Shatian’ pomelo) at early (12 wpg) and late (48 wpg) CLas infection stage were described in detail in the following section.

### Cell wall metabolism

**Figure 4:**
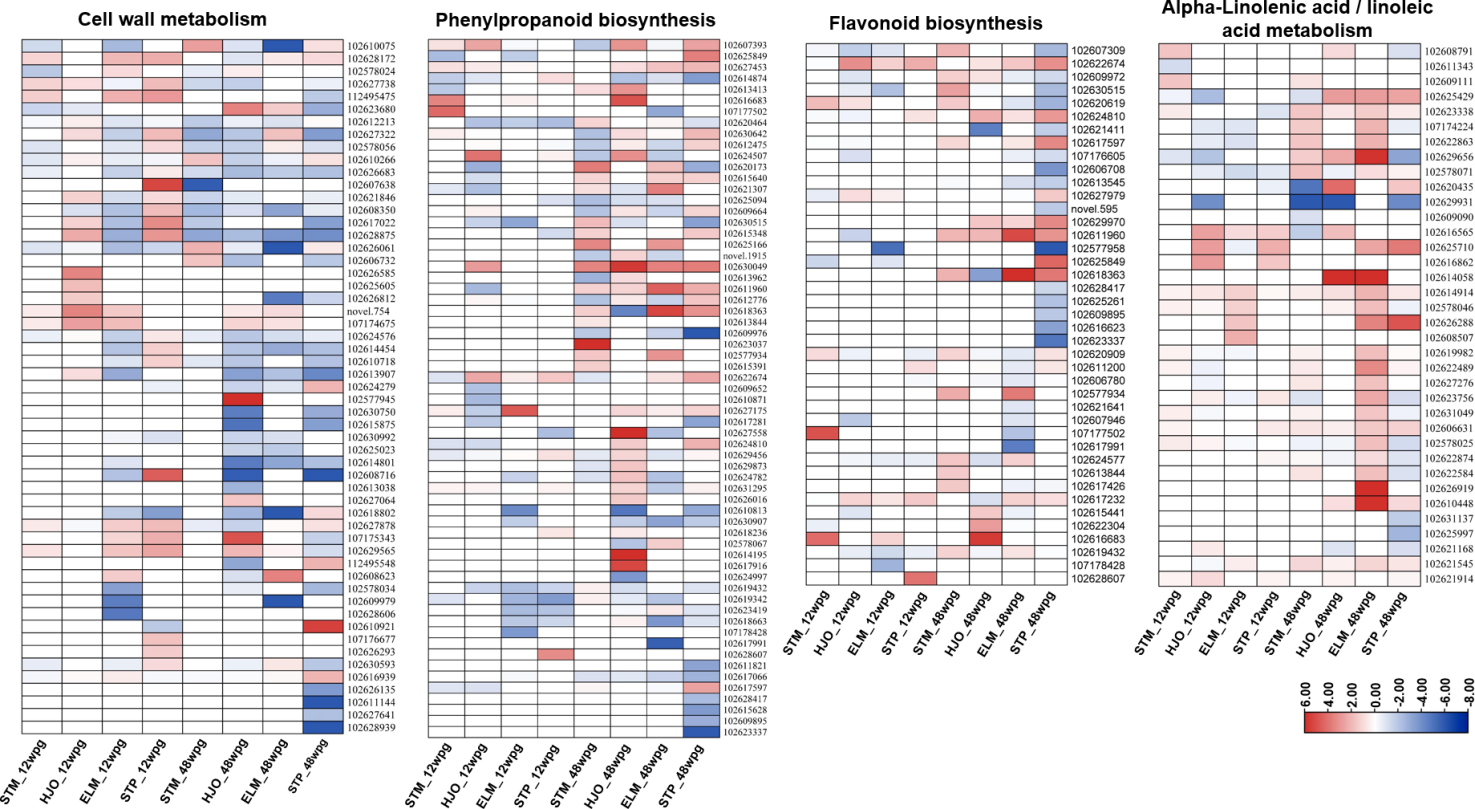
Heatmaps of differentially expressed genes (DEGs) involved in cell wall metabolism, phenylpropanoid biosynthesis, flavonoid biosynthesis and alpha-Linolenic acid/linoleic acid metabolism. STM, ‘Shatangju’ mandarin; HJO, ‘Hongjiang’ orange; ELM, ‘Eureka’ lemon; STP, ‘Shatian’ pomelo.

A total of 55 DEGs involved in cell wall metabolism were identified in four cultivars at two disease development stages (**Figure 4**, **Supplementary Table S6**). Overall, more upregulated genes involved in cell wall biogenesis, cell wall modification and organization were identified at 12 wpg than that those observed at 48 wpg in four cultivars (**Figure 4**). Particularly, 19 out of 23 DEGs were significantly upregulated in ‘Shatian’ pomelo at 12 wpg (**Supplementary Table S6**). At 12 wpg, five DEGs encoding endochitinase (102627878, 107175343, 102629565, 102628172, 112495475) were significantly upregulated in ‘Eureka’ lemon and ‘Shatian’ pomelo, while most of these genes were not significantly expressed in susceptible ‘Shatangju’ mandarin and ‘Hongjiang’ orange (**Figure 4**, **Supplementary Table S6**). In contrast, three DEGs encoding pectinesterase (102626812, 102626135, 102611144), two DEGs encoding COBRA-like protein (102628939, 102630593), and two DEGs encoding xyloglucan endotransglucosylase/hydrolase (XTH) (102609979, 102627641) were significantly downregulated in either ‘Eureka’ lemon or ‘Shatian’ pomelo, or both at 48 wpg (**Figure 4**, **Supplementary Table S6**). It was also found that genes encoded chitinase/endochitinase (102626061, 102606732, 107174675, 102629565, 107175343), pectinesterase (102610266, 102577945), XTH (102627064), COBRA-like protein (102610075) were either upregulated in ‘Shatangju’ mandarin or ‘Hongjiang’ orange at 48 wpg, whereas no significant difference of these genes was observed in tolerant ‘Eureka’ lemon and ‘Shatian’ pomelo (**Figure 4**, **Supplementary Table S6**).

### Secondary metabolism

267 DEGs involved in several biological processes of secondary metabolism were identified in four cultivars at two CLas infection stages, mainly including the phenylpropanoid biosynthesis, flavonoids biosynthesis and sesquiterpenoid and triterpenoid biosynthesis. Among these processes, the phenylpropanoid biosynthesis were most enriched in four cultivars at both 12 wpg and 48 wpg (**Supplementary Table S5**), while the flavonoids biosynthesis and sesquiterpenoid and triterpenoid biosynthesis were only enriched in ‘Eureka’ lemon and ‘Shatian’ pomelo at 48 wpg (**Supplementary Table S5**). A higher number of upregulated DEGs involved in the phenylpropanoid biosynthesis and flavonoids biosynthesis were observed at 48 wpg compared to those identified in four cultivars at 12 wpg (**Figure 4**, **Supplementary Table S5**). Especially, a burst of upregulated DEGs involved in phenylpropanoid biosynthesis was identified in ‘Eureka’ lemon at 48 wpg compared to 12 wpg (**Figure 4**, **Supplementary Table S** **5**). Several key genes involved in biosynthesis of phenylpropanoid and flavonoids were only highly induced in either ‘Eureka’ lemon or ‘Shatian’ pomelo, or both at 48 wpg, whereas the expression of these genes was not in high level or downregulated in susceptible ‘Shatangju’ mandarin and ‘Hongjiang’ orange (**Figure 4**, **Supplementary Table S7**). These DEGs included three *shikimate O-hydroxycinnamoyl transferase* (102618363, 102624810, 102617597), two *caffeoyl-CoA O-methyltransferase* (102622674, 102627979), four *caffeic acid 3-O-methyltransferase* (102630642, 102621307, 102625166, 102612776), two *vinorine synthase-like* (102625849, 102611960) and one *flavonol synthase* (102629970) (**Figure 4**, **Supplementary Tabl** **e S7**).

### Lipid metabolism

Several pathways involved in lipid metabolism, mainly including alpha-linolenic acid metabolism, linoleic acid metabolism, and cutin, suberine and wax biosynthesis, were significantly enriched in four cultivars at two stages (**Supplementary Table** **S5**). Most of DEGs involved in alpha-linolenic acid metabolism and linoleic acid metabolism were upregulated in ‘Eureka’ lemon and ‘Shatian’ pomelo at both 12 wpg and 48 wpg (**Figure 4**). Particularly, all DEGs involved in alpha-linolenic acid and linoleic acid metabolism were upregulated in ‘Eureka’ lemon at 48 wpg (**Figure 4**, **Supplementary Table S8**), indicating a strong activation of alpha-linolenic and linoleic acid metabolism in ‘Eureka’ lemon in response to CLas infection at 48 wpg. It was found that four DEGs involved in alpha-linolenic and linoleic acid metabolism, including two *linoleate 13S-lipoxygenase* (102625710 and 102626288), one *4-coumarate-CoA ligase* (102606631) and one *alpha-dioxygenase* (102610448) were only significantly upregulated in both ‘Eureka’ lemon and ‘Shatian’ pomelo at 48 wpg (**Figure 4**, **Supplementary Table** **S8**).

### Hormone-related pathways

**Figure 5:**
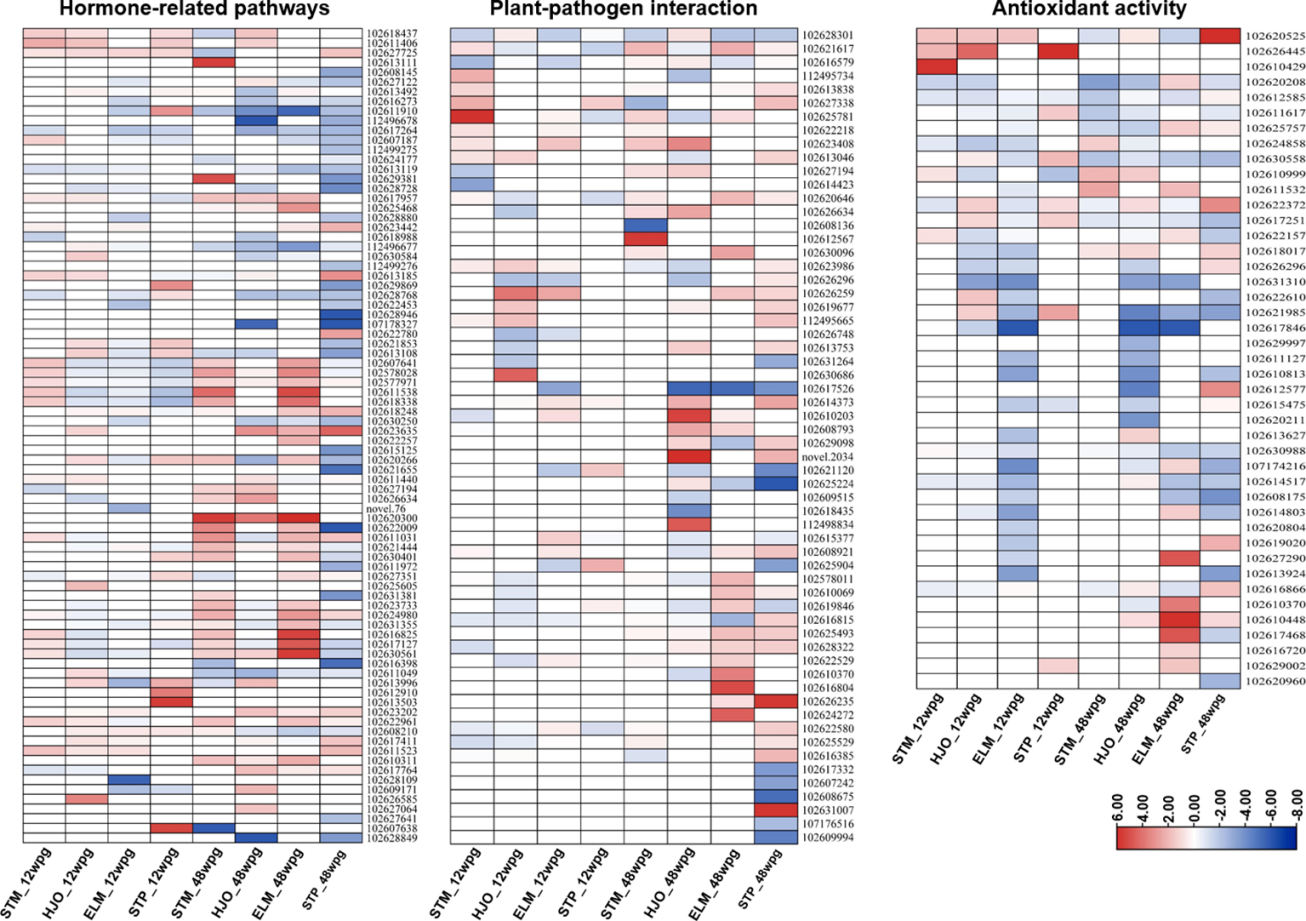
Heatmaps of differentially expressed genes (DEGs) involved in hormone-related pathways, plant-pathogen interaction and antioxidant activity. STM, ‘Shatangju’ mandarin; HJO, ‘Hongjiang’ orange; ELM, ‘Eureka’ lemon; STP, ‘Shatian’ pomelo.

A total of 80 DEGs were enriched in several hormone-related pathways, including ethylene (ET), abscisic acid (ABA), auxin (AUX) and gibberellin (GA), jasmonic acid (JA) and salicylic acid (SA) (**Supplementary Table S9**). Overall, more DEGs related to hormone pathways were identified in four cultivars at 48 wpg than those observed at 12 wpg (**Figure 5**). The ethylene metabolism was induced in ‘Shatangju’ mandarin at 12 wpg (**Figure 5**, **Supplementary Table S9**). Particularly, five DEGs involved in ethylene metabolism was only induced in ‘Shatangju’ mandarin at 12 wpg, including two ethylene-responsive transcription factor 1B (102611538, 102618338), one EIN3-binding F-box (102607641), one ethylene response 2 (102578028) and one ethylene response sensor (102577971) (**Figure 5**, **Supplementary Table S9**). In addition, three salicylic acid-binding protein 2 (SABP2) (102613996, 102612910, 102613503) were highly upregulated in ‘Shatian’ pomelo at 12 wpg (**Figure 5**, **Supplementary Table S9**), indicating SA pathway was strongly induced in ‘Shatian’ pomelo in response to CLas at early infection stage. In contrast, the auxin metabolism pathway was repressed at 48 wpg in four cultivars with most of DEGs related to auxin metabolism were downregulated (**Supplementary Table S9**). Particularly, for a total of 26 genes involved in auxin metabolism, 22 were repressed in ‘Shatian’ pomelo at 48 wpg (**Figure 5**, **Supplementary Table S9**). One auxin-induced protein AUX22-like (102607187) and one auxin-responsive protein IAA14 (102613119) were only downregulated in ‘Eureka’ lemon and ‘Shatian’ pomelo at 48 wpg (**Supplementary Table S9**). In addition, a gibberellin receptor GID1B, an important nuclear receptor for gibberellin signal transduction, was only upregulated in ‘Eureka’ lemon and ‘Shatian’ pomelo at 48 wpg (**Supplementary Table S9**).

### Plant-pathogen interaction

A total of 63 DEGs involved in the plant-pathogen interaction were identified in response to CLas infection in four cultivars at 12 wpg and 48 wpg (**Figure 5**, **Supplementary Table S1** **0**). Overall, most of DEGs related to plant-pathogen interaction in four cultivars were upregulated at both 12 wpg and 48 wpg (**Figure 5**, **Supplementary Table S** **10**). At 12 wpg, a slightly increased number of upregulated genes related to plant-pathogen interaction were identified in susceptible ‘Shatangju’ mandarin and ‘Hongjiang’ orange compared to tolerant ‘Eureka’ lemon and ‘Shatian’ pomelo (**Figure 5**, **Supplementary Table S10**). These upregulated DEGs in ‘Shatangju’ mandarin or ‘Hongjiang’ orange at 12 wpg mainly involved in cyclic nucleotide-gated ion channels (CNGCs) and cytosolic Ca^2+^ elevation, including six *cyclic nucleotide-gated ion channels* (CNGCs) (102625781, 102627338, 102626259, 112495665, 102619677, 102623986) and one *calcium-binding protein CML39-like* (102613838) (**Figure 5**, **Supplementary Table** **S10**). It was also found that three genes involved in CNGCs (102627338, 102626259, 102621120) were upregulated in either ‘Shatian’ pomelo or ‘Eureka’ lemon (**Figure 5**, **Supplementary Table** **S10**). In contrast, several genes involved in plant pathogen associated molecular pattern (PAMP)-triggered immunity (PTI) were only induced in ‘Shatian’ pomelo or ‘Eureka’ lemon at 12 wpg, including a *mitogen-activated protein kinase (MAPK) 6* (102625904), two *LRR receptor-like serine/threonine-protein kinase* (102614373, 102610203) and one *PTI1-like tyrosine-protein kinase 3* (102615377) (**Figure 5**, **Supplementary Table S10**).

A burst of upregulated DEGs involved in plant-pathogen interaction observed in infected ‘Eureka’ lemon and ‘Shatian’ pomelo at 48 wpg (**Figure 5**, **Supplementary Table** **S10**). The number of upregulated DEGs identified in ‘Eureka’ lemon (17 genes) and ‘Shatian’ pomelo (21 genes) at 48 wpg was higher than those identified in ‘Shatangju’ mandarin (8 genes) and ‘Hongjiang’ orange (12 genes) (**Figure 5**, **Supplementary Table** **S10**). DEGs involved in cyclic nucleotide-gated ion channel, calcium cell signaling pathways, leucine-rich repeat (LRR) receptor-like serine/threonine-protein kinase, WRKY transcription factor and pathogenesis-related genes transcriptional activator were strongly induced in ‘Eureka’ lemon or/and ‘Shatian’ pomelo at 48 wpg, while most of these genes were not significantly changed in ‘Shatangju’ mandarin and ‘Hongjiang’ orange at 48 wpg (**Figure 5**, **Supplementary Table** **S10**). Particularly, five DEGs involved in plant defense response were only upregulated in both ‘Eureka’ lemon and ‘Shatian’ pomelo at 48 wpg, including a *cyclic nucleotide-gated ion channel 1-like* (102626259), a *calcium-binding protein CML44* (102628322), a *pathogenesis-related genes transcriptional activator* (*PTI5*, 102625493), a *LRR receptor-like serine/threonine-protein kinase* (*At3g47570*, 102626235) and a *WRKY transcription factor 33* (102608921) (**Figure 5**, **Supplementary Table** **S10**).

### Antioxidant activity

A total of 43 DEGs involved in antioxidant activity were identified in four cultivars at two stages, including various types of peroxidase, superoxide dismutase, catalase isozyme, alpha-dioxygenase and respiratory burst oxidase (**Figure 5**, **Supplementary Tabl** **e S11**). Several types of *peroxidase* were significantly induced in ‘Shatangju’ mandarin, ‘Hongjiang’ orange and ‘Shatian’ pomelo at 12 wpg (**Figure 5**, **Supplementary Table** **S11**). Notably, a *peroxidase 51-like* (102610429) was significantly upregulated (Log_2_FC = 5.85) in ‘Shatangju’ mandarin and a *peroxidase P7-like* (102626445) was significantly activated in ‘Hongjiang’ orange (Log_2_FC = 4.36) and ‘Shatian’ pomelo (Log_2_FC = 7.39) (**Figure 5**, **Supplementary Table** **S11**). In contrast, 22 out of 23 DEGs identified in ‘Eureka’ lemon at 12 wpg were downregulated and most of these DEGs were belonging to *peroxidase* (**Figure 5**, **Supplementary Table** **S11**).

At 48 wpg, most DEGs with antioxidant activity identified in ‘Shatangju’ mandarin and ‘Hongjiang’ orange were downregulated (**Figure 5**, **Supplementary Ta** **ble S11**). However, compared to ‘Shatangju’ mandarin and ‘Hongjiang’ orange, an increased number of upregulated DEGs involved in antioxidant activity were identified in ‘Eureka’ lemon and ‘Shatian’ pomelo at 48 wpg (**Figure 5**, **Supplementary Tab** **le S11**). Three types of *respiratory burst oxidases homolog proteins* (*RBOHs*, including *protein E*, RBOHE: 102626296, *protein B*, RBOHB: 102610370 and *protein D*, RBOHD: 102616720), six *peroxidases* (102625757, 102612577, 102619020, 102627290, 102617468, 102629002, 102618017), one *L-ascorbate peroxidase* (102622372), one *catalase isozyme 1* (102616866) and one *alpha-dioxygenase 1-like* genes (102610448) were significantly upregulated in either ‘Eureka’ lemon or ‘Shatian’ pomelo, or both (**Figure 5**, **Supplementary Table S11**).

## Discussion

**Figure 6:**
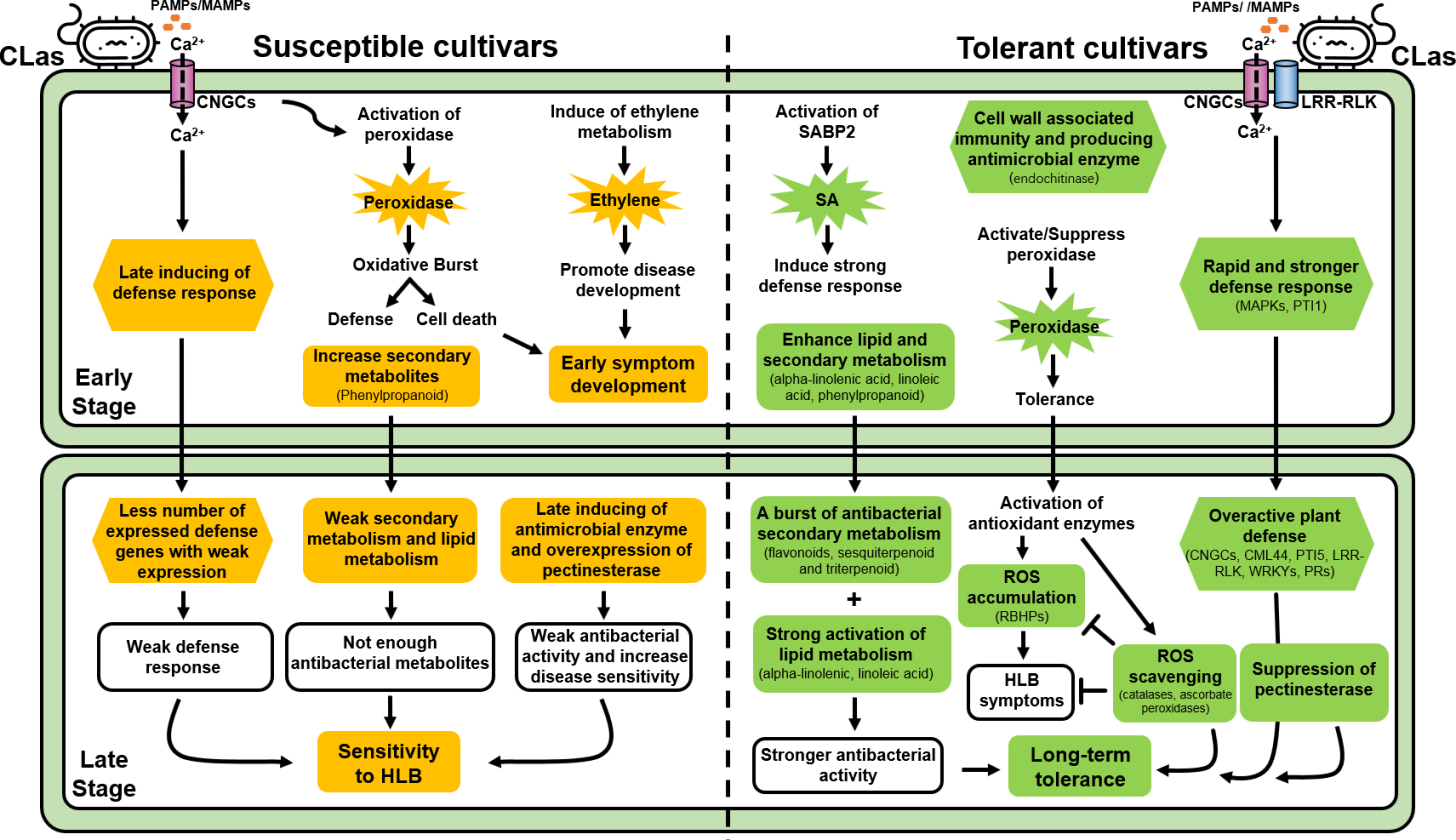
Schematic overview of important pathways of HLB-tolerant cultivars (*Citrus limon* cv. Eureka and *C. maxima* cv. Shatian Yu) and HLB-susceptible cultivars (*C. reticulata* Blanco cv. Shatangju and *C. sinensis* cv. Hongjiang Cheng) in response to “*Candidatus* Liberibacter asiaticus”.

Plant cell wall was not only known as a passive barrier upon pathogen attack, but also played an important role in plant immunity by undergoing dynamic remodeling in adaption to the pathogenic infection (Wan et al., 2021). In this study, a burst of genes involved in cell wall metabolism were observed in ‘Shatian’ pomelo at early infection stage (**Figure 4**, **Supplementary Tab** **le S6**), indicating a possible rapid cell wall associated immunity in ‘Shatian’ pomelo in response to CLas infection at early stage. Among enzymes involved in cell wall metabolism, plant endochitinase were generally upregulated by both biotic and abiotic stress and played an important role in plant resistance against distinct pathogens, including fungi and bacteria (Kasprzewska, 2003). Particularly, plant chitinases with lysozyme or lysozyme-like activity were able to cleave the peptidoglycan of bacteria (Collinge et al., 1993). Among five upregulated genes encoded endochitinase in ‘Eureka’ lemon and ‘Shatian’ pomelo, one (107175343) contained the lysozyme_like domain (cl00222) was onl y upregulated in ‘Eureka’ lemon and ‘Shatian’ pomelo at 12 wpg (**Figure 4**, **Supplementary Table** **S6**), suggested its possible role in resistance to CLas during infection at 12 wpg. However, the induce of chitinase/endochitinase in ‘Shatangju’ mandarin and ‘Hongjiang’ orange at 48 wpg indicated the antibacterial response could be delayed in susceptible cultivars. In addition to chitinase/endochitinase, the pectinesterase may increase the sensitivity of citrus plant during CLas infection. Plant pectinesterase catalyzed the de-esterification of pectin into pectate/methanol and its activity was critical for the outcome of plant-pathogen interactions by making the pectin more susceptibility to microbial pectic enzymes (Lionetti et al., 2012). Thus, the repression of pectinesterase in ‘Eureka’ lemon and ‘Shatian’ pomelo at 48 wpg may hinder the success of a subsequent CLas infection in ‘Eureka’ lemon and ‘Shatian’ pomelo at late infection stage, while the overexpression of pectinesterase genes in susceptible ‘Shatangju’ mandarin and ‘Hongjiang’ orange could increase the sensitivity to HLB and promote the disease development within plant at late infection stage (**Figure 6**).

The enhance of secondary metabolism promoted the tolerance of ‘Eureka’ lemon and ‘Shatian’ pomelo to HLB. Compared to HLB-susceptible cultivars, a burst of upregulated DEGs involved in secondary metabolism (phenylpropanoid, flavonoids, sesquiterpenoid and triterpenoid) were observed in HLB-tolerant cultivars (**Figure 4**, **Supplementary Tabl** **e S7**). Particularly, several key genes (*shikimate O-hydroxycinnamoyl transferase*, *caffeoyl-CoA O-methyltransferase*, *caffeic acid 3-O-methyltransferase*, *flavonol synthase*) involved in biosynthesis of phenylpropanoid and flavonoids were only induced in ‘Eureka’ lemon and ‘Shatian’ pomelo (**Figure 4**, **Supplementary Tab** **le S7**). Plant secondary metabolites played an important role in plant defense response to pathogens infection with its antimicrobial activity (Zaynab et al., 2018). Among secondary metabolites, phenylpropanoids were widely distributed in the plant and played critical roles in plant development by serving as essential components of cell walls, or in response to abiotic or biotic stress, such as wounding, high light/UV radiation and pathogen infection (Korkina, 2007). Flavonoids were a family of plant-derived compounds with well-known antibacterial activity (Cushnie and Lamb, 2011). Previous study found that the *shikimate O-hydroxycinnamoyl transferase* was actively expressed in vascular tissues and played a key role in phenylpropanoids and lignin biosynthesis (Hoffmann et al., 2004). Both *caffeoyl-CoA O-methyltransferase* and *caffeic acid 3-O-methyltransferase* were involved in disease resistance to multiple pathogens by controlling the level of lignin and other metabolites of phenylpropanoid pathway and suppressing the programed cell death (Guo et al., 2001; Yang et al., 2017). In addition, the *flavonol synthase* catalyzed the conversion of dihydroflavonols to flavonols and played a central role in flavonoid biosynthesis (Liu et al., 2021). Therefore, the upregulation of genes involved in phenylpropanoid biosynthesis and flavonoids biosynthesis in ‘Eureka’ lemon and ‘Shatian’ pomelo at late CLas infection stage could contribute to their long-term tolerance to CLas (**Figure 6**).

Plant antimicrobial lipids, mainly including fatty acids and monoglycerides, had been recognized as broad-spectrum antibacterial agents by lysing bacterial cell membrane and inhibiting bacterial growth through a range of mechanisms (Alves et al., 2020). In this study, the strong activation of alpha-linolenic acid and linoleic acid metabolism was observed in two HLB-tolerant cultivars, particularly the ‘Eureka’ lemon, at both early and late infection stage compared to ‘Shatangju’ mandarin and ‘Hongjiang’ orange (**Figure 4**, **Supplementary Ta** **ble S8**). Both alpha-linolenic and linoleic acid were unsaturated fatty acid and had been found to exhibit antimicrobial activity against bacteria and fungi (Lee et al., 2002; Kusumah et al., 2020), which indicated they could be involved in plant defense during pathogen infection. A recent study showed that the alpha-linolenic acid metabolism was the only pathway enriched in citrus transgenic trees expressing *Arabidopsis thaliana NPR1* (AtNPR1), which showed potential resistance to HLB in field (Qiu et al., 2020). Overexpression of alpha-linolenic acid and linoleic acid metabolism suggested they should play an important role in the tolerance of ‘Eureka’ lemon and ‘Shatian’ pomelo during CLas infection (**Figure 6**).

Hormone was the central regulators of plant development and found to be involved in the plant defense against various pathogens infection (Verma et al., 2016). Most genes involved in ethylene metabolism was uniquely upregulated in susceptible ‘Shatangju’ mandarin at early infection stage (**Figure 5**, **Supplementary Ta** **ble S8**). Ethylene can be induced by the pathogen invasion and showed the discrepancy of the double signaling function in disease resistance in studies of various plant-pathogen interactions (Lund et al., 1998; van Loon et al., 2006). Normally, the accumulation of ethylene in plant promoted the disease development simply through its acceleration of ripening or senescence (Panter and Jones, 2002). Previous study found that genes involved in the ethylene pathway were also upregulated in CLas-infected susceptible sweet orange (Fan et al., 2012). The activation of ethylene metabolism in ‘Shatangju’ mandarin at early infection stage may promote the HLB symptoms development, which contributed to the earlier presence of symptoms in ‘Shatangju’ mandarin compared to tolerant cultivars (**Figure 1**, **Table 1**). In addition, three *salicylic acid-binding proteins* were strongly induced in tolerant cultivar ‘Shatian’ pomelo at 12 wpg (**Figure 5**, **Supplementary Tabl** **e S8**). SA was an essential plant defense hormone that promoted immunity in response to both biotic and abiotic stresses (Koo et al., 2020; Peng et al., 2021). The *SABP2* was a well-characterized protein essential for establishment of methyl salicylate (MeSA)-mediated systemic acquired resistance (SAR), which provided the enhanced immunity to a secondary pathogen infection in tissues distal to the site of primary infection (Liu et al., 2011; Soares et al., 2022). A recent study showed that the overexpression of *SABP2* was able to enhance tolerance to HLB in transgenic citrus by reducing HLB symptoms and repressing CLas titer in a low level (Soares et al., 2022). Therefore, the activated expression of *SABP2* genes could contribute to suppression of HLB symptoms development and CLas propagation in ‘Shatian’ pomelo at early infection stage (**Figure 6**). In addition, previous studies also found that SA was able to inhibit the auxin signaling pathways as part of the plant defense mechanism (Wang et al., 2007 **;** Kazan and Manners, 2009). The global suppression of auxin signaling pathway in tolerant ‘Shatian’ pomelo at 48 wpg could be also caused by the significant upregulation of SA pathway, indicated that the SA-mediated plant defense may play important role in tolerance of ‘Shatian’ pomelo during CLas infection (**Figure 6**).

The different defense response pattern to CLas infection played an important role in susceptibility and tolerance to HLB at early infection. At 12 wpg, genes involved in CNGCs and cytosolic Ca^2+^ elevation were upregulated in susceptible cultivars group, while genes involved in PTI were only upregulated in tolerant cultivars group (**Figure 5**, **Supplementary Tab** **le S10**). Plant CNGCs played important roles in the pathogen signaling cascade and facilitated cytosolic Ca^2+^ elevation in response to pathogen and PAMP signals (Ma and Berkowitz, 2011; Moeder et al., 2011). The transient cytosolic Ca^2+^ influx had been demonstrated to be a core event for triggering PTI (Boudsocq et al., 2010; Dodd et al., 2010), the first layer of plant immunity for microbial perception and restricting pathogen proliferation (Chisholm et al., 2006; Jones and Dangl, 2006). Based on different defense response pattern to CLas infection at early infection stage, the tolerant cultivars exhibited a rapid and stronger defense response to limit the CLas growth after infection, while the susceptible cultivars could be delayed in defense response at early infection stage. In addition, a burst of upregulated DEGs involved in plant defense pathways was identified in tolerance of ‘Eureka’ lemon and ‘Shatian’ pomelo compared to ‘Shatangju’ mandarin and ‘Hongjiang’ orange at 48 wpg (**Figure 5**, **Supplementary Ta** **ble S10**). Taken together with the moderate symptoms and lower CLas concentration observed in ‘Eureka’ lemon and ‘Shatian’ pomelo during the whole infection stage, the rapid and stronger defense response at early CLas infection stage and overactive of plant defense reaction at late CLas infection stage could provide the long-term tolerance to HLB by suppressing the disease symptom development and CLas propagation in tolerant cultivars during CLas infection (**Figure 6**).

Plant peroxidases participated in various physiological process, such as lignification, wound healing, auxin catabolism and defense mechanisms against pathogen infection (Hiraga et al., 2001; Cosio and Dunand, 2009). In this study, several types of peroxidase were induced in ‘Shatangju’ mandarin, ‘Hongjiang’ orange and ‘Shatian’ pomelo at 12 wpg, while most *peroxidases* were suppressed ‘Eureka’ lemon at 12 wpg (**Figure 5**, **Supplementary Tabl** **e S11**). Previous study found that the induce at transcript and protein level of multiple peroxidases in citrus during CLas infection was related to the activation of plant defense responses (Franco et al., 2020). In addition, peroxidase was also able to generate high reactive oxygen species (ROS), which induced the programmed cell death of plant (Cosio and Dunand, 2009). HLB was thought as a pathogen-triggered immune disease due to the accumulation of CLas-triggered ROS in phloem-enriched bark tissue, which following caused the systemic cell death of companion and sieve element cells (Ma et al., 2022). The diverse function of *peroxidase* indicated that the upregulation of *peroxidase* in susceptible ‘Shatangju’ mandarin and ‘Hongjiang’ orange at early stage could be mainly involved in defense during CLas infection by inducing oxidative burst, which further caused systemic cell death in infected citrus plant. However, the heterogeneous expression of *peroxidase* between ‘Eureka’ lemon and ‘Shatian’ pomelo at early infection stage suggested that the function of *peroxidase* may not be the major characteristics of HLB tolerance at early stage (**Figure 6**). Additionally, DEGs involved in antioxidant activity were mainly suppressed in ‘Shatangju’ mandarin and ‘Hongjiang’ orange at late infection stage (**Figure 5**, **Supplementary Tabl** **e S11**), which indicated a weak antioxidant defense response at late infection stage. In contrast, an increased number of antioxidant-related genes and ROS related genes (*RBOHs*, *peroxidases*, *L-ascorbate peroxidase*, *catalase*) were upregulated in ‘Eureka’ lemon and ‘Shatian’ pomelo at late infection stage (**Figure 5**, **Supplementary Tabl** **e S11**). Among RBOHs, the RBOHD was known as a central driving force of ROS signaling in plant cells in reaction to pathogen associated molecular patterns during plant-pathogen interaction (Torres et al., 2002). The RBOHD-dependent ROS accumulation triggered by CLas infection was able to cause the cell death of phloem tissue of citrus plant (Ma et al., 2022). However, the suppressing of ROS-mediated cell death caused by CLas infection was able to mitigate HLB symptoms in infection plants (Ma et al., 2022). Both catalases and ascorbate peroxidases were two main H_2_O_2_ scavenging enzymes in plants (Sofo et al., 2015). In this study, the activation of catalases and ascorbate peroxidases in tolerant cultivars could help to alleviate the oxidative stress caused by ROS, which in turn to reduce HLB symptoms at late infection stage. Therefore, the induce of genes involved in ROS signaling and scavenging process suggested they could not only enhance the tolerance to CLas infection but also limited the HLB symptom in ‘Eureka’ lemon and ‘Shatian’ pomelo at late infection stage (**Figure 6**).

## Conclusion

Grafting-based CLas infection assay showed *C*. *limon* and *C*. *maxima* were tolerance to HLB, while *C*. *reticulata* Blanco and *C*. *sinensis* showed sensitive to HLB. Comparative transcriptomic analysis indicated that the stronger responses in SA-mediated immune, PTI, cell wall associated immunity, endochitinase, phenylpropanoid and alpha-linolenic/linoleic lipid metabolism, occurred in HLB-tolerant *C*. *limon* and *C*. *maxima* may play important roles against CLas infection at early infection stage. In contrast, the induce of oxidative burst and ethylene metabolism, as well as the delayed defense response may lead to the early HLB symptom development in susceptible *C*. *reticulata* Blanco and *C*. *sinensis* at early infection stage. In addition, the overactive plant defense, stronger antibacterial activity and the activation of ROS scavenging genes could contribute to the long-term tolerance to HLB in *C. limon* and *C. maxima* at late infection stage. However, the weak defense response and antibacterial secondary metabolism, as well as the induce of pectinesterase could be main reason of for HLB sensitivity in *C. reticulata* Blanco and *C. sinensis* at late infection stage.

## Data availability statement

The original contributions presented in the study are publicly available. This data can be found here: https://www.ncbi.nlm.nih.gov/bioproject/PRJNA941950.

## Author contributions

CG, CL, XD and ZZ conceived and designed the experiments. CG, CL, ZL, YL, JL, JG, JM, FF, CW and ZZ performed the experiments. CG, CL, JM and ZZ contributed to the bioinformatic and statistical analyses. CG and CL prepared the figures/tables and drafted the manuscript. CG, CL, XD and ZZ revised the manuscript. All authors contributed to the article and approved the submitted version.
