# Supplementary material for: Comparative transcriptome profiling of susceptible and tolerant citrus species at early and late stage of infection by “Candidatus Liberibacter asiaticus”
Source: Front Plant Sci. 2023 Jun 14;14:1191029. doi: 10.3389/fpls.2023.1191029 (PMC10301834; doi:10.3389/fpls.2023.1191029)
Supplement: Supplementary file 2 [file DataSheet_2.pdf]

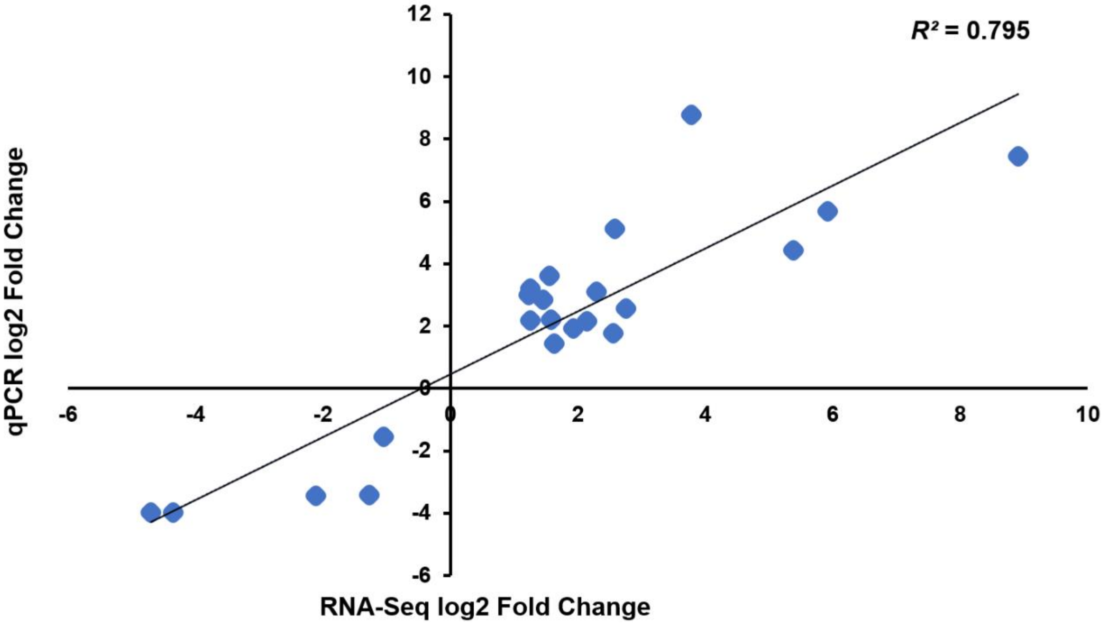

Figure S1. Correlation of 15 selected DEGs gene expression between RNA-seq and qPCR validation in four cultivars.

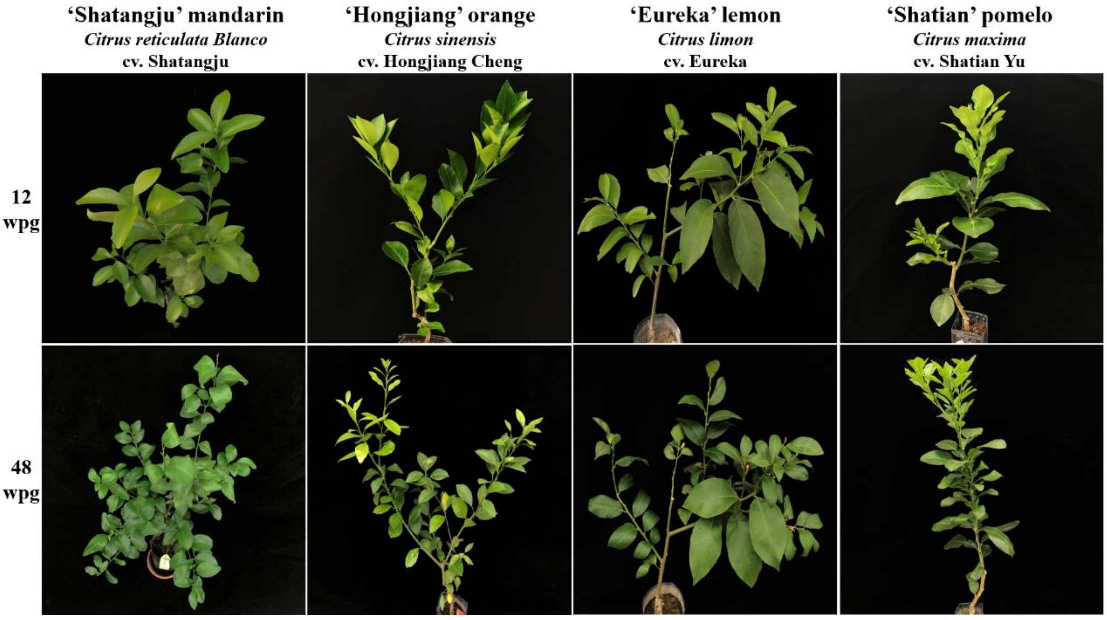

Figure S2. The representative citrus plants (mock-grafted) of four cultivars selected for RNA-Seq analysis after 12 and 48 weeks post-grafting.

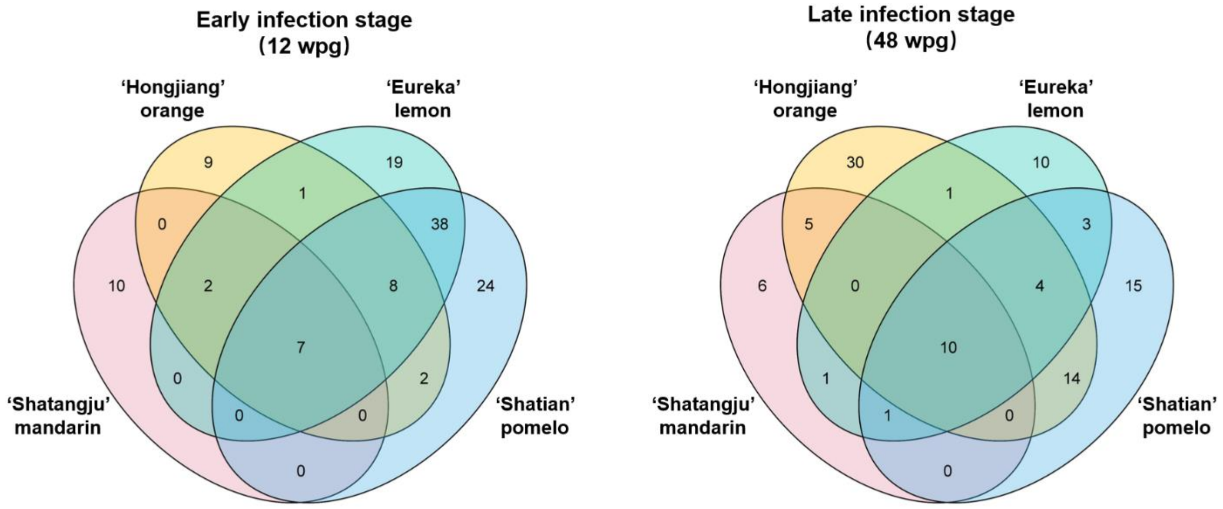

Figure S3. GO terms cluster of DEGs in four cultivars at early and late disease development stage.
